# Personal and professional challenges confronted by hospital staff following hurricane sandy: a qualitative assessment of management perspectives

**DOI:** 10.1186/s12873-016-0082-5

**Published:** 2016-05-05

**Authors:** Andrea M. Morris, Karen A. Ricci, Anne R. Griffin, Kevin C. Heslin, Aram Dobalian

**Affiliations:** Department of Health Policy and Management, Fielding School of Public Health, University of California, Los Angeles (UCLA), Los Angeles, CA USA; US Department of Veterans Affairs, Veterans Emergency Management Evaluation Center (VEMEC), North Hills, CA USA; Department of OB/GYN Research, Baystate Medical Center, Springfield, MA USA; US Department of Health and Human Services, Center for Delivery, Organization, and Markets (CDOM), Agency for Healthcare Research and Quality, Rockville, MD USA; School of Nursing, UCLA, Los Angeles, CA USA

**Keywords:** Disaster response, Recovery, Hospital, Professional duty, Veterans, US Department of Veterans Affairs

## Abstract

**Background:**

Adequate hospital staffing during and after a disaster is critical to meet increased health care demands and to ensure continuity of care and patient safety. However, when a disaster occurs, staff may become both victim and responder, decreasing their ability and willingness to report for work. This qualitative study assessed the personal and professional challenges that affected staff decisions to report to work following a natural disaster and examined the role of management in addressing staff needs and concerns.

**Methods:**

Semi-structured interviews were conducted with individuals who filled key management roles in the United States Department of Veterans Affairs New York Harbor Healthcare System’s response to Superstorm Sandy and during the facility’s initial recovery phase. All interviews were audio recorded and transcribed. Three major themes were identified: 1) Barriers to reporting (“Barriers”), 2) Facilitators to reporting (“Facilitators”), and 3) Responses to staff needs and concerns (“Responses”). Atlas.ti 7.1.6 software program was used for the management and analysis of the transcripts.

**Results:**

Results indicated that staff encountered several barriers that impeded their ability to report to work at mobile vans at the temporarily nonoperational Manhattan campus or at two other VA facilities in Brooklyn and the Bronx in the initial post-Sandy period including transportation problems, personal property damage, and communication issues. In addition, we found evidence of facilitators to reporting as expressed through descriptions of professional duty. Our findings also revealed that management was aware of the challenges that staff was facing and made efforts to reduce barriers and accommodate staff affected by the storm.

**Conclusions:**

During and after a disaster event, hospital staff is often confronted with challenges that affect decisions to report for work and perform effectively under potentially harsh conditions. This study examined barriers and facilitators that hospital staff encountered following a major natural disaster from the management perspective. Insights gained from this study can be used to inform future disaster planning and preparedness efforts, and help ensure that there is adequate staffing to mount an effective response when a disaster occurs, and to recover from its aftermath.

## Background

Public health emergencies and disasters place an enormous strain on health care systems and have the potential to disrupt the delivery of health services and compromise patient care. Having adequate staffing during and after a disaster is critical to hospital surge capacity, i.e., a hospital’s ability to “expand quickly beyond normal services” to meet increased health care demands [1], and to ensure continuity of care and patient safety [2–5]. However, a comprehensive review of more than 30 years of literature on emergency health care workers’ responses to emergencies and disasters suggests that it is unrealistic to assume that all health care workers will report to work during or shortly after an event [6]. When disasters occur, staff may become both victims and responders, thus complicating their decisions regarding whether to report for duty. As a result, it is important to identify factors that affect reporting decisions in order to ensure there is adequate staffing to mount an effective response and meet the surge in demand.

Qureshi and colleagues distinguish between two concepts that affect decisions to respond to work during a disaster: ability to respond and willingness to respond [7]. ‘Ability’ refers to one’s capacity to report (i.e., is available and has the means to report) whereas ‘willingness’ refers to a personal choice to report [3, 7, 8]. Although the two concepts are similar, Qureshi and colleagues [7] point out “even if one is fully able, he or she might still not be willing to report to work.” Conversely, one may be willing, but lack the ability to report to work. Previous research has shown that hospital staff is often confronted with multiple barriers that impact their ability and willingness to report to work following a disaster [3, 7]. Typical barriers that affect the ability to report during a disaster include transportation problems and obligations to care for children, elders, pets, and other dependents [3, 6, 7, 9, 10]. In terms of willingness, fear and concern for self, family, and pets, as well as personal health problems have been reported as barriers [3, 7, 9, 11]. Additional factors shown to influence decisions to report include perceived emergency preparedness of the organization, perceived importance of one’s role during a disaster, and the strength of an individual’s sense of professional duty [11–16]. In addition, prior experience with disasters has been shown to influence hospital evacuation and disaster response decisions [14, 17].

Although there is substantial literature on decisions to report to work during a disaster, the majority of studies have been based on quantitative data drawn from hypothetical/scenario-based surveys to examine staff ability and willingness [3, 7, 10, 12, 18], which may not represent actual decisions made during a disaster event. Qualitative studies may allow for a more detailed description of staff needs and concerns during a disaster; however, most studies have focused on the employee perspective to identify factors associated with decisions to report [9, 11, 14]. To our knowledge, previous work has not examined the role of executive and middle management decision makers in identifying and reducing barriers to reporting. Research describing the management perspective could inform the development of more effective strategies to address staff needs and concerns in advance, which may reduce barriers to reporting, and thus, mitigate the potential adverse impact of staff shortages on patient care [14]. In this study, we examine the impact of personal and professional challenges on staff response and recovery efforts, using qualitative data from interviews with individuals who filled key management roles in the United States Department of Veterans Affairs (VA) during Superstorm Sandy (“Sandy”) - the most devastating natural disaster ever to hit NYC and the second-costliest hurricane in U.S. history - which made landfall on the New York/New Jersey area on October 29, 2012 [19–21].

Massive flooding across Lower Manhattan led to the closure of five acute care hospitals, including the Manhattan campus of the VA New York Harbor Healthcare System (NYHHS). VA NYHHS chose to evacuate its Manhattan VA Medical Center (VAMC) in advance of the storm rather than shelter-in-place, transferring patients to neighboring VA facilities or discharging some to the community. Due to extensive flood damage, the Manhattan facility remained closed for a few months post-Sandy. During this time, Manhattan VAMC staff reported to VA Brooklyn and Bronx facilities.

## Methods

### Participants and sampling

For exploratory purposes, a qualitative approach using semi-structured interviews was chosen. Interviews were conducted 12 weeks post-Sandy with staff from the Manhattan campus of the VA NYHHS and from VA New York/New Jersey Veterans Integrated Service Network 3 (VISN 3), the regional network that includes NYHHS, who participated in disaster response efforts. Potential interview respondents were identified through existing contacts in VA at local, regional and national levels, and through VA documents (e.g., After Action Reports (AARs), NYHHS online directory). Stratified purposive sampling techniques were used to select individuals who had filled key roles in NYHHS’s response to Sandy. The analytic sample consists of 32 participants, including senior leaders (executive managers and clinicians in senior administrative roles), clinical decision makers (nurses, physicians, and social workers), and individuals from emergency management, transport, and support services (e.g., food, laundry). Oral consent was obtained from respondents to be both interviewed and recorded. Respondents could decline to be interviewed, end the interview at any point, or participate in an interview but not be recorded in whole or part. The VA Greater Los Angeles Healthcare System’s Institutional Review Board (IRB) approved the study protocol.

### Data collection

Interviews were conducted by at least two members of the research team and lasted 60–90 min. The interview guide was developed based on an instrument used in a prior study on VA nursing home evacuations after Hurricanes Katrina and Rita [22], a hospital evacuation tool by Schultz and colleagues [23], and the hospital evacuation literature [24–27]. Interview respondents were asked about successes, challenges, and lessons learned.

### Data analysis

All interviews were audio recorded and transcribed *verbatim*. The Atlas.ti 7.1.6 software program was used for the management and analysis of the transcripts. Two members of the project team (KR and AG) read the transcripts and highlighted sections of text using preliminary codes based on the interview guide and hospital evacuation literature. This process resulted in the identification of 55 quotes related to the impact of Sandy on hospital staff. An additional project team member (AM) then conducted a pile sorting exercise to identify major themes among the quotes. Three theme definitions were developed from the exercise: 1) Barriers to reporting (“Barriers”), 2) Facilitators to reporting (“Facilitators”), and 3) Responses to staff needs and concerns (“Responses”). Theme definitions and the quotes were first discussed with a separate team member (KH) and then provided to an additional team member (AG), who conducted an independent pile sort, reviewing the quotes and assigning each one to one of the 3 a priori themes. Agreement between the results of the pile sorting by these two authors was estimated for each theme definition using the Kappa statistic. Kappa for “Barriers” was found to be .55, indicating moderate agreement; .69 for “Facilitators,” indicating substantial agreement; and .84 for “Responses,” indicating almost perfect agreement [28]. Disagreements in coding were resolved through discussion.

## Results

Respondents described several instances of personal and professional challenges that impacted the ability and willingness of staff to report to work during the first 3 months following Sandy. Respondents in management positions also described efforts to address staff needs and concerns while balancing managerial responsibilities.

### Barriers to reporting

Our findings showed that staff encountered several barriers that impeded their ability to report to work. The most commonly reported barrier was transportation problems. In the aftermath of the storm, much of Lower Manhattan remained under water, major roadways were closed, and the public transit system effectively shut down. Moreover, the Manhattan VA facility was itself flooded and mostly nonoperational at the time the interviews for this study were conducted. Although some staff reported to work at mobile vans at the temporarily closed campus, most staff reported to work at one of two other VA facilities in Brooklyn and the Bronx. As a result, many staff members faced difficulties getting to work for the first several days following Sandy. One respondent stated, “I was completely crippled for several days myself, I had no way to get anywhere, once Manhattan went down, there was no public transportation; I didn’t have a vehicle.” Although transportation conditions gradually improved in the weeks following Sandy, several respondents commented on the impact that transportation issues had on staffing during initial response efforts. As a key NYHHS decision-maker stated: “There was no public transportation. They [staff managers] had a very hard time.... They had no staffing. They couldn’t get staff in here.”

In addition to transportation barriers, our findings suggest that personal property damage or loss contributed to staffing issues. According to several comments, there were a number of staff that was unable to come to work because their “homes were wiped out.” A NYHHS Emergency Manager received notification from one of the medical centers that, “…they were facing huge staff shortages for two reasons, one is that I’ve lost my house and I’m not coming to work, and secondly, I didn’t lose my house, but I can’t get to work.”

Several respondents also noted that communication issues posed a challenge to reporting after the storm. The day before Sandy struck, one respondent described sending email messages and announcements to staff alerting them to the Manhattan VAMC evacuation and closure in anticipation of the storm. However, Sandy caused widespread power outages that lasted several days, and in some areas weeks, which hindered efforts to communicate with staff after the storm and led to some confusion about where staff was supposed to report. One senior leader described efforts to communicate with staff during this time period: “…we hoped that those who could communicate with each other would get the messaging out that we needed, which was that employees were to report to their nearest facility.”

### Facilitators to reporting

Although respondents described several barriers to staff reporting to work, we also found evidence of facilitators to reporting as expressed through descriptions of professional duty. For example, several respondents described the overwhelming response by staff who wanted to help and reported for duty “despite their own personal hardships.” While describing some of the challenges that staff was dealing with, one senior leader shared:

There were so many stories floating around. There was one person who couldn’t find her parents for a long time, but she was here. There was another story about a nursing assistant saying, “All the clothes I own, I’m wearing,” but he was here. You know, there were people who just make your left ventricle swell, they were just unbelievable. Not that everybody was terrific and not that there weren’t people, ‘I can’t believe I have to come all the way over here,’ but most people really understood, these are our veterans and we’re taking care of them.

Our findings suggest that a strong sense of duty was an important factor in the decision to report to work. However, it was also evident that personal circumstances and concerns played a role in the decision process, with several comments speaking to challenges balancing personal and professional obligations. For example, one senior leader shared, “…a lot of the staff are victims, too, in this and so they’re trying to manage multiple responsibilities and priorities,” with the top priority being the health and safety of their own families. Another respondent described the dilemma faced by emergency management staff when deciding whether or not to report to work: “When do you leave your own place and say ‘I should come in’ or say ‘well, my house is going to flood and I’ve got to watch my kids, so what do I do?’”

### Management responses to staff needs and concerns

Our findings showed that management readily acknowledged the personal challenges that staff was dealing with and made efforts to accommodate staff impacted by the storm. For example, one clinical decision maker stated: “…we had a lot of staff that got hit very hard. They lost their homes, they lost their car, and they had no place to live. You see them. You ask them what’s going on. Any information you can give them, you give it to them. You make them feel as though you care about them as people, not just as workers.” Another respondent described efforts to obtain leave time for staff who were directly impacted by the storm: “we really pushed hard where we could for getting people time off, getting authorized absence for people who were in situations that really they were genuinely crippled…”

In addition, leadership at both the NYHHS level and the VISN level described instances where VA was able to anticipate and address staff needs and concerns. For example, post-Sandy, staff was instructed to report to the closest facility. In recognition of the confusion this would likely cause, a ‘welcoming center’ was set up at every facility in order to assign staff a role. However, comments from several respondents suggested that there were also limitations to the support that management could provide. When asked “what can the VA do” to assist staff who were having a difficult time getting to work, a senior leader replied: “…we’ve got authority to move them from one location to the hospital, like if they carpooled and went to a parking lot, we can pick them up but you can’t pick them up at home and bring them in. So there are some limits to what you can do.” Despite these regulatory challenges, efforts were made to reduce transportation barriers. For example, one senior leader described putting in a shuttle bus system to help transport staff members who were able to report to the Manhattan campus over to the other VAMCs.

Although there were numerous descriptions of efforts to help staff, respondents in management positions described the challenge of trying to balance employee needs and management responsibilities. For example, while acknowledging the personal losses suffered by some staff members, a senior leader expressed concern about mounting an effective disaster response with fewer staff, “We had a number of people… that… literally lost everything and in the meanwhile, you have to kind of cobble together a response.” With these issues in mind, another respondent discussed the impact that employee preparedness has on the ability of staff to report during disaster response: “…if they don’t have daycare, they don’t have all the family care plan things that we talk about should be in place, they can’t come in.”

## Discussion

The present study examined the challenges faced by hospital staff following a major natural disaster from the management perspective. Results suggest that there are several factors that affect decisions to report for duty during post-disaster response and early recovery efforts. Our findings also indicated that management was aware of the challenges that staff was dealing with and proactively made efforts to provide assistance to staff when possible. The ability to forecast challenges and address barriers in advance may allow management to better plan and prepare for predictable problems and increase the likelihood of being able to ensure adequate staffing to provide timely access to care following a natural disaster [14].

This study is unusual in that it discusses issues regarding reporting to work at facilities other than the facility that was itself rendered temporarily nonoperational by the disaster. Nevertheless, the findings are often similar to the findings of prior studies. For example, consistent with previous research [3, 7, 12], our findings showed that staff encountered several barriers following Sandy including transportation problems and personal property damage. Establishing a workforce readiness plan that includes provisions such as temporary housing and travel assistance could serve to mitigate these barriers and increase facilitators to reporting [29].

Our findings also showed that communication issues served as a barrier to reporting to work. Communication plays a critical role during disaster response in providing emergency responders with pertinent information about appropriate actions to take. However, during a disaster, communication channels often fail, making it difficult to transmit and receive information. As reported by several respondents, insufficient information during and immediately after Sandy led to confusion about when and where staff was supposed to report for work. Simple tools such as telephone notification cascades, provided telephone or text service is available, can strengthen communication capacity by providing staff with a protocol to be implemented in the event of a disaster.

Although mitigating barriers is important for increasing staff’s ability to report for work in a disaster, an individual must be both willing and able to respond in order to ensure that there is adequate staffing to meet pre-existing and increased demand for health services. In the present study, we found evidence of staff willingness to report as expressed through descriptions of professional duty. Previous research suggests that decisions to report to work are related to an individual’s sense of professional duty and are positively influenced by a caring connection with the organization [11, 14–16]. For example, a qualitative study found that hospital staff who perceived their organization as having a caring and supportive culture was more willing to report during a fire disaster [14]. In this study, this kind of connectedness was indicated by numerous reports of dedicated staff members overcoming barriers and reporting to work “despite personal hardships,” and through descriptions of management’s efforts to reduce barriers and support staff needs. Davidson and colleagues [14] suggest that connectedness with an organization is cultivated prior to the disaster and further defined by leadership actions during a disaster or emergency. There also is evidence to suggest that a supportive organizational culture is strongly associated with employee satisfaction - with management practices and leadership in the workplace identified as important drivers of satisfaction [30, 31]. Therefore, efforts to improve employee satisfaction and build resiliency may strengthen one’s sense of professional duty and dedication to the organization, increasing the likelihood that the employee will report to work.

Effective emergency preparedness is critical to mounting a successful disaster response and for ensuring minimal disruptions to patient care. While workplace preparedness is important for a successful response [11, 12], personal disaster preparedness planning is also necessary for ensuring staff readiness after a disaster [2]. Previous research suggests that when a disaster strikes, staff may be confronted with competing personal and professional duties [3, 14]. In line with this, several respondents described the personal challenges that staff was dealing with during the response and early recovery period. Respondents also described their own challenges with managing multiple responsibilities during this time. Incorporating employee preparedness efforts into existing workplace preparedness practices, such as working with employees to develop and maintain a personal disaster plan, may help to reduce the tension between personal and professional responsibilities and facilitate willingness to report [3, 9]. In addition, effective employee preparedness may also allow management to better balance the needs of employees with management responsibilities.

There are several limitations to this study. First, the data were collected from a purposive sample. As a result, the findings may not be reflective of VA management views beyond the key leaders involved in this one disaster. In addition, the data represent the experience of a single facility within VA and the single region within which that facility resides, thereby limiting the generalizability of the findings to other facilities. It should also be noted that study participants often provided third-person accounts of staff experiences, which may not be reflective of the actual experiences of staff. Although the results are in strong accordance with prior research findings, the inclusion of additional frontline staff in future work would allow researchers to assess and compare disaster response decisions from multiple perspectives. Finally, this study assessed management perspectives of challenges faced by staff following a natural disaster. Given that response efforts and associated challenges may differ depending on the type of event, future work should investigate staff response efforts from the management perspective for other types of disasters and emergencies.

## Conclusion

In the past decade, we have witnessed a dramatic increase in the number and severity of weather-related disasters [29]. The ability to meet increased demand for health services during a disaster event requires having available staff to meet those needs [2, 4]. However, when a disaster occurs, staff may encounter personal and professional challenges that decrease their ability and willingness to respond. This study examined barriers and facilitators that hospital staff encountered following a major natural disaster from the management perspective. Our findings suggest that proactively addressing staff needs and concerns can help mitigate barriers, strengthen professional duty, and increase willingness to report. In addition, improving workplace and personal preparedness efforts can help ensure that there is adequate staffing to mount an effective response when a disaster occurs.

## Ethics approval and consent to participate

The VA Greater Los Angeles Healthcare System’s Institutional Review Board (IRB) approved the study protocol. Oral consent was obtained from respondents to be both interviewed and recorded.

## Consent for publication

Not Applicable

## Availability of data and materials

Making the interview data publically available is not practicable in a deidentified fashion and would therefore violate the ethics approval. Subject to this limitation, data will be made available to interested researchers to the fullest extent permissible by contacting the VA Greater Los Angeles Institutional Review Board. The interview guide is provided.

## Abbreviations

AAR: After Action Report
IRB: Internal Review Board
NYHHS: New York Harbor Healthcare System
VA: United States Department of Veterans Affairs
VAMC: VA Medical Center
VISN: Veterans Integrated Service Network
